# L-lysine Functionalized Mesoporous Silica Hybrid Nanoparticles for pH-Responsive Delivery of Curcumin

**DOI:** 10.3390/pharmaceutics15061631

**Published:** 2023-05-31

**Authors:** Madhappan Santhamoorthy, Vanaraj Ramkumar, Kokila Thirupathi, Lalitha Gnanasekaran, Vanitha Karuppannan, Thi Tuong Vy Phan, Seong-Cheol Kim

**Affiliations:** 1 School of Chemical Engineering, Yeungnam University, Gyeongsan 38541, Republic of Korea; santham83@yu.ac.kr (M.S.); sriram27@yu.ac.kr (V.R.); 2 Department of Physics, Government Arts and Science College for Women, Karimangalam, Dharmapuri 635111, Tamil Nadu, India; kokila66@gmail.com; 3 Departamento de Ingeniería Mecánica, Facultad de Ingeniería, Universidad de Tarapacá, Avda. General Velásquez 1775, Arica 1000007, Chile; lalitha88@gmail.com; 4 Department of Physics, Bannari Amman Institute of Technology, Sathyamangalam, Erode 638401, Tamil Nadu, India; vanithak@bitsathy.ac.in; 5 Center for Advanced Chemistry, Institute of Research and Development, Duy Tan University, 03 Quang Trung, Hai Chau, Da Nang 550000, Vietnam; 6 Faculty of Environmental and Chemical Engineering, Duy Tan University, 03 Quang Trung, Hai Chau, Da Nang 550000, Vietnam

**Keywords:** mesoporous silica nanoparticles, drug delivery, pH-stimuli, surface modification, L-lysine

## Abstract

Stimuli-responsive controlled drug delivery systems have attracted the attention of researchers in recent decades due to their potential application in developing efficient drug carriers that are responsive to applied stimuli triggers. In this work, we present the synthesis of L-lysine (an amino acid that combines both amine and carboxylic acid groups in a single unit) modified mesoporous silica nanoparticles (MS@Lys NPs) for the delivery of the anticancer bioactive agent (curcumin, Cur) to cancer cells. To begin, mesoporous silica hybrid nanoparticles (MS@GPTS NPs) with 3-glycidoxypropyl trimethoxy silane (GPTS) were synthesized. The L-lysine groups were then functionalized onto the mesopore channel surfaces of the MS@GPTS NPs through a ring-opening reaction between the epoxy groups of the GPTS and the amine groups of the L-lysine units. Several instrumental techniques were used to examine the structural properties of the prepared L-lysine-modified mesoporous silica nanoparticles (MS@Lys NPs). The drug loading and pH-responsive drug delivery behavior of MS@Lys NPs were studied at different pH levels (pH 7.4, 6.5, and 4.0) using curcumin (Cur) as a model anticancer bioactive agent. The MS@Lys NPs’ in vitro cytocompatibility and cell uptake behavior were also examined using MDA-MB-231 cells. The experimental results imply that MS@Lys NPs might be used in cancer therapy as pH-responsive drug delivery applications.

## 1. Introduction

Cancer is a disease that affects millions of people worldwide. It is a group of diseases that involve abnormal cell multiplication and spread throughout the body [1]. Cancer may affect any region of the body and be caused by several factors such as genetics, lifestyle, and environmental exposure [2]. Cancer therapies include surgery, chemotherapy, radiation therapy, immunotherapy, and targeted therapy. Although there is no efficient remedy for cancer at the moment, early detection and treatment can significantly improve outcomes for many cancer patients [3].

Chemotherapy is a type of cancer treatment in which potent drugs are used to eradicate cancer cells. It is effective against certain cancers because it targets aggressively proliferating cells [4]. Chemotherapy can be administered on its own or in combination with other therapies such as surgery and radiation therapy [5,6]. Although chemotherapy has been used for many years [7], novel therapies that are more effective and have fewer adverse effects are being developed. Curcumin, a polyphenol derived from the turmeric plant’s root, has been used in traditional medicine [8]. It has been extensively explored in recent years due to its potential health benefits. Curcumin is well known for its anti-inflammatory and antioxidant properties, which may help reduce inflammation associated with chronic conditions such as arthritis, diabetes, and heart disease [9,10,11]. Curcumin may also possess neuroprotective effects, which may aid in the prevention of brain disorders such as Alzheimer’s and Parkinson’s disease [12,13].

Controlled drug delivery systems are conceived to release drugs in a controlled manner over time [14]. Among the techniques employed are encapsulation, microspheres, liposomes, and nanoparticles. Controlled drug delivery systems outperform traditional techniques in various ways, including increased bioavailability, targeted distribution, fewer side effects, and improved patient compliance [15]. This approach has the potential to be utilized to treat chronic diseases such as cancer and diabetes. Stimuli-responsive drug delivery is a unique approach that regulates drug release by using external stimuli such as light, pH, temperature, or a magnetic field, among other things [16,17,18]. It has the potential to transform the way drugs are administered to patients by enabling accurate and targeted drug delivery at the appropriate time and location. This method offers multiple benefits compared to conventional drug administration systems, including improved safety, efficacy, and patient compliance [19].

Because they have the potential to target particular cells or tissues, nanoparticles have become an important tool for drug delivery [20]. Nanoparticles enable them to penetrate deeper into the body and more precisely target specific locations. The nanoparticles are considerably more stable than the organic molecules in the biological media, which makes them appropriate for long-term therapy. As a result, nanoparticles are an excellent drug delivery method that is both efficient and safe. Mesoporous silica nanoparticles (MSNs) are increasingly being employed for drug delivery applications due to their unique properties [21,22]. Since MSNs are porous, they can more effectively encapsulate and transport drugs than ordinary nanoparticles. They also have a large surface area, which allows for increased drug-loading capacity and targeted drug administration [23]. MSNs are also biocompatible, making them appropriate for therapeutic usage. As a result, they are an attractive candidate for drug delivery systems because they may transport drugs directly to specific locations with minimal toxicity to surrounding cells or tissue [24].

Surface modification of mesoporous silica nanoparticles with efficient functional units such as ligand groups or organic groups might improve their efficacy in certain applications. Various biomolecules including L-histidine and disulfide bonds are employed for surface modification/integration of mesoporous silica nanoparticles for controlled drug delivery applications due to their biocompatibility [25,26]. L-lysine is an amino acid that is essential for protein synthesis and collagen synthesis, as well as for wound healing [27]. The chemical structure of L-lysine consists of two amino groups and a carboxylic group in a single unit [28]. Because of the presence of amine and carboxylic acid groups in L-lysine, they could serve as drug-binding sites when functionalized in/onto mesoporous silica nanoparticles. Amine and carboxylic acid groups on the surface of mesoporous silica may improve the adsorption of various drugs or biomolecules. The modification of amino acids onto the silica surfaces improves the adsorption efficiency of the organic molecules. Various reports have demonstrated that the synthesis of poly-L-lysine-grafted silica nanoparticles would be of considerable interest because the high density of cationic charges on the surface would have many applications including gene delivery. For example, Li et al. used poly-L-lysine-modified mesoporous silica nanoparticles for plasmid DNA delivery [29]. Lunn et al. modified the silica surfaces of L-alanine by surface-initiated polymerization of L-alanine onto the amine-functionalized silica materials [30]. Similarly, Qiao et al. reported the synthesis of poly-L-lysine functionalized mesoporous silica nanoparticles for gene delivery [31]. However, this approach of functionalization procedure resulted in a significant reduction of porosity of the silica particles and pore blockage at high biomolecule loading.

This work focuses on the preparation of a pH-responsive drug delivery system based on mesoporous silica nanoparticles functionalized with L-lysine, and loaded with the bioactive agent, curcumin (Cur). Firstly, mesoporous silica was selected as the inorganic support for drug loading. Secondly, L-lysine was functionalized onto the silica surfaces as the hosting sites to control the release of the drug/bioactive agents. We choose different pH values (pH 7.4, 6.5, and 4.0) to verify the release behavior at various pH conditions. In a physiological pH environment, the L-lysine functional units, the encapsulated drug/bioactive agent are firmly bound and thus prevent the premature release of cargo. In an acidic environment, the loaded cargo was released from the carrier system because of the electrostatic repulsive force between the protonated L-lysine and the cargo molecules. The bioactive agent, curcumin-loaded MS@Lys/Cur NPs showed enhanced release of loaded cargo under acidic conditions (pH 6.5 and 4.0) than under physiological conditions (pH 7.4). The literature reports revealed that various mesoporous silica materials were modified with different polymer-based amino acids such as poly-L-lysine and poly-L-alanine for biomolecule delivery. However, the L-lysine groups immobilized mesoporous silica nanoparticle system for controlled drug delivery of drug/bioactive agents are rarely reported. Among them, L-lysine groups are considerably interesting because they have two amine groups and one carboxylic acid group in a single molecule and the terminal amine groups could be utilized for further modification process.

In the present work, 3-glycidoxypropyltrimethoxysilane (GPTS)-incorporated mesoporous silica nanoparticles (MS@GPTS NPs) were synthesized by co-condensation method by using the cetyltrimethylammonium bromide (CTAB) as a templating agent. Moreover, the L-lysine groups (which include both amine and carboxylic acid groups in a single unit) were functionalized onto the mesopore channel surfaces of the prepared MSN@GPTS NPs via a ring-opening reaction between the GPTS epoxy groups and the amine groups of the L-lysine units. Several instrumental techniques were used to investigate the structural properties of the prepared L-lysine-modified mesoporous silica nanoparticles (MS@Lys NPs). The pH-responsive drug delivery behavior of MS@Lys NPs was studied at three different pH levels such as pH 7.4, 6.5, and 4.0, respectively, using curcumin (Cur) as a model anticancer bioactive agent. The MS@Lys NPs’ in vitro cytocompatibility and cell uptake behavior was also examined using MDA-MB-231 cells. The present approach demonstrates the utilization of amino acid as a functional unit to modify the silica nanoparticles for storing and controlled release of various drug/bioactive agents under different pH stimuli conditions in cancer therapy.

## 2. Materials and Methods

### 2.1. Chemicals and Reagents

Hexadecyltrimethylammonium bromide (98%), (3-glycidoxypropyl)trimethoxysilane (GPTS, 98%), tetraethylorthosilicate (TEOS, 98%), ammonium nitrate (98%), ethanol (96%), L-lysine (98%), curcumin (Cur, 98%), and 3-[4,5-dimethylthiazol-2-yl]-2,5-diphenyltetrazolium bromide (MTT) were purchased from Sigma-Aldrich Company (Saint Louis, MO, USA). Deionized water was used to prepare all the solutions. All the reagents were used without further purification.

### 2.2. Preparation of GPTS-Incorporated Mesoporous Silica Nanoparticles (MS@GPTS NPs)

The GPTS precursor integrated mesoporous silica nanoparticles were prepared using sol-gel hydrolysis and co-condensation technique. To do this, 1.5 g of CTAB was suspended in 200 mL of deionized water and stirred magnetically. To this, 3 g of ammonia solution was added and agitated for 30 min to make a clear solution. Following that, a premixed solution (20:80 mol% of GPTS:TEOS in 5 mL ethanol) was slowly added to the reaction flask containing CTAB solution. The suspension solution was magnetically stirred for 12 h at 45 °C and 24 h at 80 °C.

**Scheme 1:**
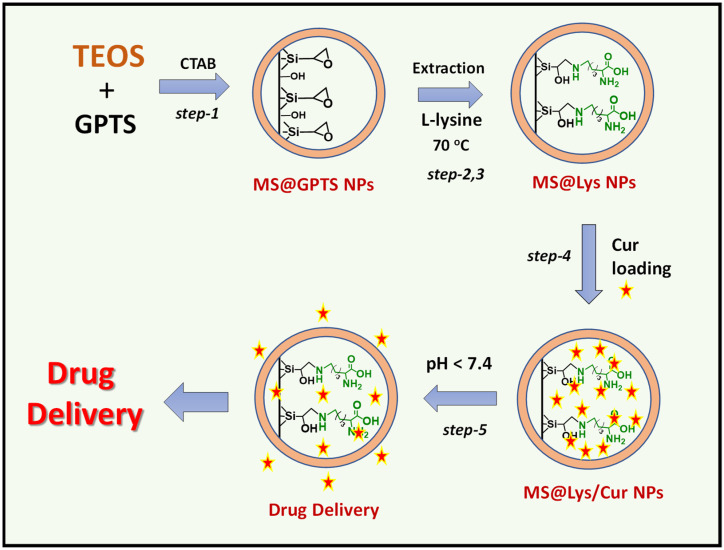
Schematic representation for the preparation of MS@GPTS NPs (step-1); surfactant extraction and L-lysine functionalities modified MS@Lys NPs (step-2,3); curcumin loading into the MS@Lys NPs (step-4); pH-responsive drug release behavior of the MS@Lys/Cur NPs NPs (step-5), respectively.

The precipitate was rinsed with deionized water and ethanol before drying at 60 °C. Lastly, the CTAB was extracted using an ethanolic ammonium nitrate solution (1 g NH_4_NO_3_ in 50 mL ethanol), which was repeated three times for the complete removal of CTAB. The CTAB removed sample was named MS@GPTS NPs) (Scheme 1, step-1,2) [32].

### 2.3. Preparation of L-Lysine Modified Mesoporous Silica Nanoparticles (MS@Lys NPs)

To synthesize L-lysine-modified mesoporous silica nanoparticles, 0.5 g of MS@GPTS NPs were dispersed in 100 mL of ethanol-water mixture (EtOH: water, 40:60) and sonicated for 5 min to generate a homogeneous dispersion. To this purpose, about 0.2 g (0.0136 mol) of L-lysine was dissolved in 10 mL deionized water and added to the reaction mixture while magnetic stirring was performed. The reaction mixture was allowed to react for 12 h at 70 °C [33]. Finally, the suspension was filtered, rinsed with deionized water and ethanol, and vacuum-dried overnight at 50 °C. The L-lysine modified sample was labeled as MS@Lys NPs (Scheme 1, step-3).

### 2.4. Characterization

X-ray diffraction (XRD) pattern was recorded on a Bruker AXN X-ray diffractometer using Cu Kα radiation (λ = 1.5418 Å). Images of scanning electron microscopy (SEM) were obtained using a JEOL 6400 microscope set to 20 kV. Fourier transform infrared (FTIR) spectra were obtained using the KBr pellet technique on a JASCO FTIR 4100 apparatus at 5000 psi hydraulic pressure. Field emission transmission electron microscopy (FETEM, JEOL 2010) images were obtained at an accelerating voltage of 200 kV. A Nova 4000 e surface area analyzer was used to perform N_2_ adsorption-desorption studies. The Barrett–Joyner–Halenda (BJH) technique was used to calculate the pore size from the adsorption branch. On a Malvern Zetasizer Nano-ZS (Malvern Instruments), the surface charge and particle size in suspension were measured. Thermogravimetric analysis (TGA) was performed on a Perkin-Elmer Pyris Diamond thermogravimetric analyzer with a heating rate of 10 °C/min under N_2_ atmosphere. Images of fluorescence microscopy were captured using a Leica (TCS-SP2) fluorescence microscope.

### 2.5. Curcumin Loading into the MS@Lys NPs

Curcumin was used as a model anticancer bioactive agent to investigate the drug loading efficiency and release behavior of the prepared MS@Lys NPs under varied pH (pH 7.4, 6.5, and 4.0) conditions. The MS@GPTS NPs were utilized as a control sample for comparison. For the loading procedure, 0.2 g of MS@Lys NPs or MS@GPTS NPs were placed in a 100 mL round-bottomed flask containing 50 mL ethanol, and 50 mg of Cur in 5 mL ethanol was added. The suspension was then sonicated for 5 min and magnetically stirred for 2 h. The slurry was then centrifuged, washed with ethanol (1 mL), and the resultant Cur-loaded sample was vacuum-dried overnight at 40 °C. The Cur-loaded sample was named as MS@Lys/Cur NPs (Scheme 1, step-4). The concentrations of the initial drug solution and the supernatant solution collected after adsorption were used to determine the drug loading efficiency. Eventually, the drug loading efficiency was determined as follows. Drug loading efficiency (%) = [C_i_ − C_e_]/C_i_ × 100. C_i_ represents the initial drug concentration. C_e_: unloaded drug (supernatant solution after washing). Therefore, the loading efficiency of Cur into the MS@Lys NPs was around ~68%, whereas the control sample MS@GPTS NPs showed only approximately ~32% Cur loading efficiency. The drug loading content was also estimated by using the following equation. Drug loading (%) = [Wt. of drug − Wt. of NPs] × 100. The content of loaded Cur into the MS@Lys/Cur NPs and the MS@GPTS/Cur NPs were determined to be approximately 34 wt.% and 16 wt.%, respectively.

### 2.6. In Vitro Drug Release Study

To investigate the in vitro drug release performance of the MS@GPTS/Cur NPs and MS@Lys/Cur NPs, the drug release efficacy was evaluated at varied pH (pH 7.4, 6.5, and 4.0) settings using the dialysis bag method [34]. To carry out this experiment, about 50 mg of Cur-loaded sample was suspended in a dialysis bag with a molecular weight cutoff of 10 kDa and immersed in a 10 mL phosphate buffer saline (PBS) solution at 37 °C with magnetic stirring. To maintain the sink condition, about 1 mL of the release medium was taken out and replaced with an equivalent amount of buffer solution at predetermined time intervals. A UV-vis spectrometer set to 435 nm was used to examine the released Cur (Scheme 1, step-5).

### 2.7. In Vitro Cytotoxicity Assay

The MTT test was used to assess the cytotoxicity of the prepared MS@GPTS NPs, MS@Lys NPs, Cur loaded MS@GPTS/Cur NPs, MS@Lys/Cur NPs, and free Cur. MDA-MB-231 cells were grown in 96-well plates for 24 h in a DMEM medium. The existing medium was then discarded, and a new medium containing varying concentrations (25 to 200 μg/mL) of MS@Lys NPs, MS@Lys/Cur NPs, and free Cur was added into each well. At each concentration, three wells were used and labeled appropriately. Following 24 h of incubation at 37 °C and 5% CO_2_, the existing medium was changed to a fresh medium containing 10% MTT solution (5 mg/mL) and incubated for another 5 h. Finally, the medium was discarded and 25 μL fresh DMSO was added to each well. Finally, the absorption values were measured at 570 nm. Cell viability was calculated as follows. Cell viability (%) = [Abs_570_ (treated cells)]/[Abs_570_ (control cells)] × 100 where Abs_570_ is the absorbance value at 570 nm.

### 2.8. Cell Uptake Study

MDA-MB-231 cells were cultured in a 6-well plate containing DMEM media for 12 h at 37 °C under 5% CO_2_ and 95% air atmosphere to investigate the in vitro cell absorption efficiency of the prepared MS@Lys/Cur NPs. The MDA-MB-231 cells were then treated to 10 μg/mL of MS@Lys NPs and Cur loaded MS@Lys/Cur NPs, respectively. The sample-treated cells were cultured for an additional 5 h before being fixed with a 4% paraformaldehyde solution. The cells were then rinsed with a cold PBS solution and observed under a fluorescence microscope.

### 2.9. Hemolysis Assay

To study the hemolysis assay, a citrated porcine blood sample was used to collect the red blood cells (RBC). About 500 μL of diluted RBCs were added into the vials containing MS@Lys NPs sample (50 mg). Further, 100 μL saline and Triton-X, respectively, were used as negative and positive controls, and the samples were incubated for 2 h at 37 °C. Then, the samples were centrifuged at 3500 rpm for 15 min. The supernatant solution was collected and analyzed using a microplate reader (570 nm). The hemolysis percentage was determined using the following equation. Hemolysis (%) = [(OD_sample_ − OD_negative control_)/(_ODpositive control_ − OD_negative control_)] × 100.

### 2.10. Statistical Analysis

The one-way ANOVA method was used to evaluate statistical significance; *p* < 0.05 was considered statistically significant. The data were presented using the mean value ± standard deviation (SD).

## 3. Results and Discussion

### 3.1. Structural Characteristics of the MS@GPTS NPs and MS@Lys NPs

**Figure 1:**
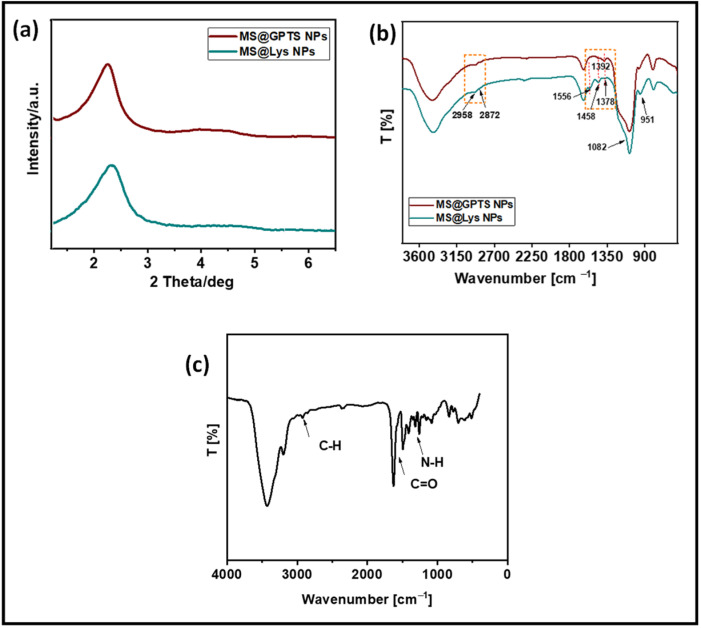
(**a**) Low-angle XRD patterns; (**b**) FTIR spectra of MS@GPTS NPs and MS@Lys NPs, respectively. (**c**) FTIR spectrum of pure L-lysine monomer.

To describe the formation of mesostructured arrangements of prepared MS@GPTS NPs and L-lysine modified MS@Lys NPs, a low-angle X-ray diffraction analysis technique was used. As shown in Figure 1a, MS@GPTS NPs exhibit a characteristic diffraction peak at 2θ = 2.25° (100) demonstrating the formation of ordered mesoporous structure in the material. Nevertheless, following the surface functionalization of L-lysine groups in the mesopore channels, the diffraction peak intensity of the MS@Lys NPs decreased slightly which is due to the pore filling of surface-modified L-lysine groups into the mesopore channels of the MS@Lys NPs. From the XRD results it could be confirmed that the existence of surface-functionalized L-lysine units within the mesopore channels and the mesoporous structure order is not considerably disturbed during the surface modification of L-lysine groups [35].

FTIR analysis was used to verify the existence of organic moieties in mesoporous silica materials. The FTIR spectra of MS@GPTS NPs and MS@Lys NPs are shown in Figure 1b. The characteristic peaks for MS@GPTS NPs at 1082 cm^−1^ and 951 cm^−1^, as shown in Figure 1b, may be ascribed to the stretching bands of Si-O-Si and surface silanol (Si-OH) groups Furthermore, the typical vibration bands at 2872 cm^−1^ and 2958 cm^−1^ show the C-H stretching of the alkyl carbon chain, suggesting the presence of GPTS units in the silica walls [36,37]. Following surface functionalization of L-lysine groups, the sample MS@Lys NPs exhibited a stretching vibrational peak at 1562 cm^−1^ for the carboxylic acid’s carbonyl -C=O vibrations, and a stretching band at 1458 cm^−1^ for the amine groups present in the L-lysine units [38]. The stretching peak intensity at 1358 cm^−1^ was reduced after reacting the amine part of L-lysine with the epoxy part of GPTS onto the MS@GPTS NPs, and a new peak appeared at 1458 cm^−1^ in the MS@Lys NPs, confirming that the L-lysine units were functionalized onto the surface of the MS@Lys NPs [39]. Figure 1c shows the FTIR spectrum of L-lysine molecules in which the stretching band at 2856 cm^−1^ and 2923 cm^−1^ indicates the C-H stretching of the alkyl carbon chain, and the bands at 1423 cm^−1^ and 1596 cm^−1^ indicate the stretching of N-H, C=O and C-OH groups present in the L-lysine molecules.

**Figure 2:**
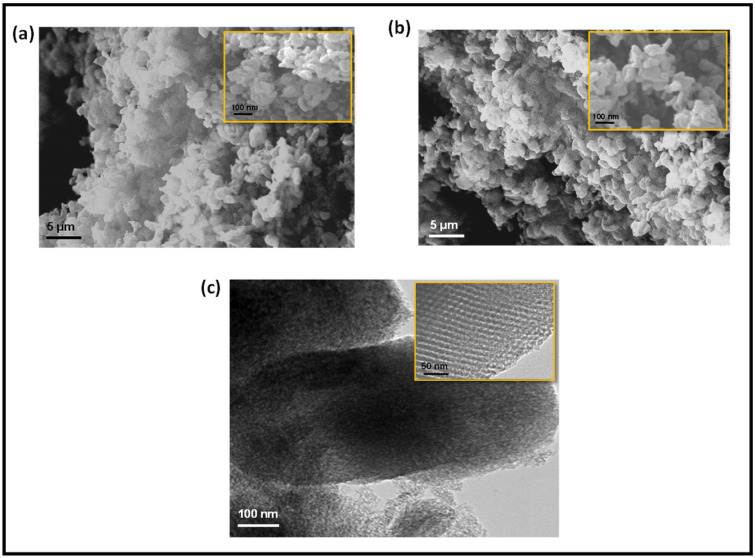
SEM images of (**a**) MS@GPTMS NPs, and (**b**) MS@Lys NPs, respectively. TEM images of MS@Lys NPs (**c**).

SEM images were used to examine the particle size and shape of the prepared MS@GPTS NPs and MS@Lys NPs. As observed in Figure 2a,b, the MS@GPTS NPs and MS@Lys NPs displayed (inset images) short rod-shaped particles with more cluster-like aggregation. As shown in Figure 2b (inset image), the MS@Lys NPs had an irregular shape and an average particle size of approximately 200–250 nm. TEM was used to investigate the formation of mesopore channels in the MS@Lys NPs. The TEM images of the MS@Lys NPs, as shown in Figure 2c, revealed the production of rod-like particles as well as the existence of clear mesopore channels (inset image), indicating the presence of mesoporous materials, and the mesoporous structure is not altered during the L-lysine surface functionalization process.

**Figure 3:**
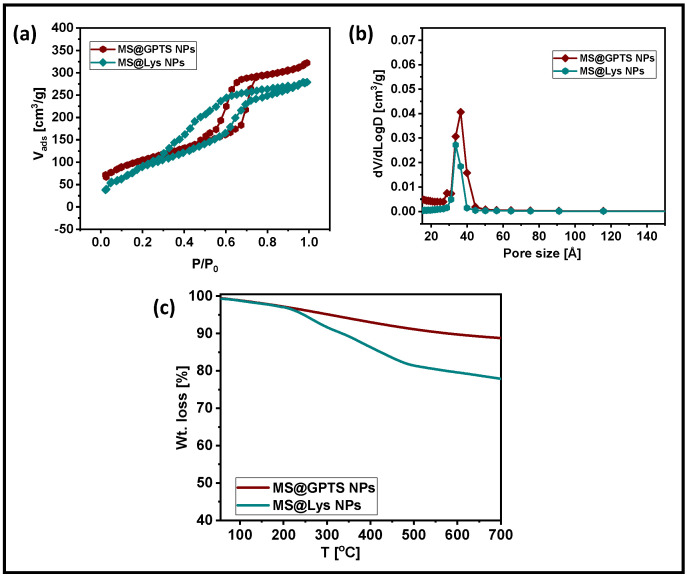
(**a**) Nitrogen adsorption–desorption isotherm curves and (**b**) pore size distribution curves, and (**c**) TG analysis curves of MS@GPTMS NPs, and MS@Lys NPs, respectively.

The nitrogen adsorption–desorption analysis and pore size distribution curves of MS@GPTS NPs and MS@Lys NPs samples are shown in Figure 3a,b. Figure 3a shows that both samples possessed type IV with hysteresis, indicating the formation of mesoporous materials. The BET surface area, pore size, and mesopore volume of the MS@GPTS NPs samples were determined to be 642 m^2^/g, 3.6 nm, and 0.042 cm^3^/g, respectively.

On the other hand, the surface functionalized MS@Lys NPs showed slightly decreased surface area, pore size, and pore volume approximately 527 m^2^/g, 3.3 nm, and 0.034 cm^3^/g, respectively. The MS@Lys NPs have lower mesopore characteristics than the MS@GPTS NPs, confirming the presence of surface-functionalized L-lysine units inside the mesopore channels of the MS@Lys NPs (Table 1) [40].

The organic functional contents in the MS@GPTS NPs and L-lysine functionalized MS@Lys NPs are estimated by thermogravimetric analysis. As shown in Figure 3c, both the MS@GPTS and MS@Lys NPs, respectively, showed a trace amount of initial weight loss (approximately 1.89 wt.%) in the temperature range at 100 °C due to the evaporation of physisorbed moisture and solvent. Next, the MS@GPTS sample showed a cumulative weight loss of about 12.4 wt.% in the temperature range from 101 °C to 650 °C, which indicates a decomposition of the quantitative amount of integrated GPTS precursors in the pore walls of the MS@GPTS NPs. Similarly, the MS@Lys NPs showed an initial weight loss of about 1.9 wt.% at 100 °C for physisorbed moisture and a collective weight loss of approximately 21.5 wt.% in the temperature range from 101 °C to 650 °C, suggesting the decomposition of surface functionalized L-lysine groups onto the mesopore surface of the MS@Lys NPs. From the TG analysis date, it could be estimated that approximately 9.1 wt.% of L-lysine was functionalized onto the mesoporous silica surface during the L-lysine modification process. Further weight loss occurring above 650 °C might be due to the decomposition of inorganic residues at higher temperatures [41].

**Figure 4:**
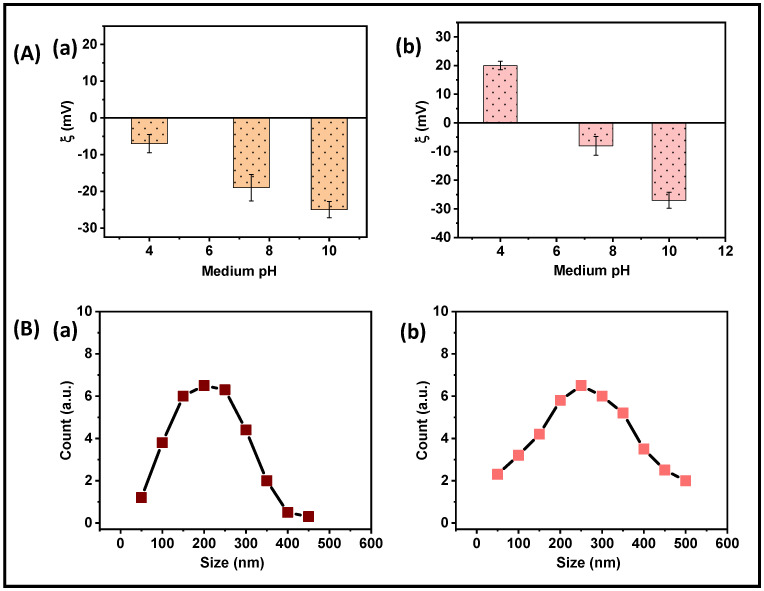
(**A**) Zeta potential analysis; (**B**) particle size distribution analysis of (**a**) MS@GPTMS NPs, and (**b**) MS@Lys NPs, respectively.

The surface charge of the GPTS-integrated MS@GPTS NPs and the L-lysine modified MS@Lys NPs was evaluated using ζ-potential analysis under varied pH settings (pH 4, 7.4, and 10). As seen in Figure 4(Aa), the MS@GPTS NPs had a ζ-potential of around −7 ± 2.58 mV, −20.4 ± 1.43 mV, and −26.1 ± 3.16 mV at pH 4, 7.4, and 10, respectively. Nevertheless, following L-lysine modification, the MS@Lys NPs exhibited a positive ζ-potential of around +18.3 ± 3.62 mV at pH 4, which may be attributed to amine group protonation Figure 4(Ab). The presence of negatively charged carboxylic acid groups in the L-lysine functionalities, on the other hand, increased the ζ-potential value to negative charge by around −10.6 ± 2.17 mV and −27.1 ± 2.62 mV at pH 7.4 and 10, respectively. The charge regulation may be explained based on the balance of the deprotonation of carboxyl groups (pKa ~2.2) and ammonium groups (pKa ~9.1 and 10.8) of the L-lysine groups. L-lysine exhibits a pKa value of about ~9.0. This is attributed to the fact that each L-lysine molecule has a carboxyl group and two amine groups that are protonated at a low pH leading to the overall charge being positive. At higher solution pH, the carboxyl groups lose the hydrogen, and becomes negatively charged. The results of the ζ-potential study confirm that the L-lysine functional units were functionalized effectively in the mesopore surface of the MS@Lys NPs. Dynamic light scattering (DLS) analysis was used to determine the particle size distribution of MS@GPTS NPs and MS@Lys NPs. As shown in Figure 4(Ba,Bb), both MS@GPTS NPs and MS@Lys NPs had particle sizes ranging from 50 to 500 nm, with an average particle size of 200–250 nm.

### 3.2. In Vitro Drug Loading/Release Study

**Figure 5:**
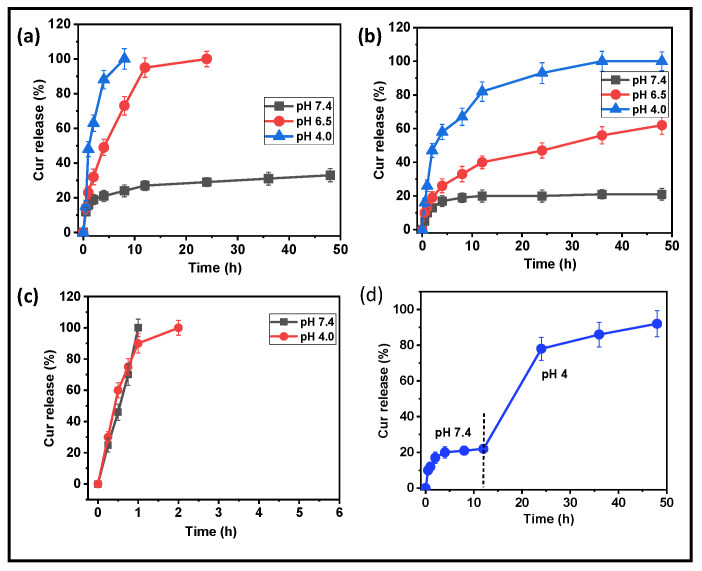
(**a**) In vitro drug release from the (**a**) MS@GPTS/Cur NPs; and (**b**) MS@Lys/Cur NPs, respectively, at different pH conditions. (**c**) In vitro release behavior of free Cur at different pH conditions. (**d**) pH-induced Cur release behavior from the MS@Lys/Cur NPs.

The drug loading and in vitro drug release behavior of the prepared MS@Lys NPs were determined using curcumin, a polyphenol-based natural drug molecule having potential anti-inflammatory and antitumor properties. Because the L-lysine molecule has both amine and carboxylic acid groups in a single molecule and can effectively interact with therapeutic molecules such as curcumin, it was utilized to functionalize the mesopore channels of the MS@GPTS NPs. As a result, it is anticipated that the L-lysine functionalized MS@Lys NPs would increase drug loading efficiency. Under physiological pH (pH 7.4) and acidic pH (pH 6.5 and 4.0) conditions, the in vitro pH-responsive release behavior of the Cur loaded MS@GPTS/Cur NPs and MS@Lys/Cur NPs was determined. PBS buffer with varied pH levels was prepared and utilized to simulate the Cur release performance in vitro. Figure 5a,b displays the cumulative release of Cur from the MS@GPTS/Cur NPs and MS@Lys/Cur NPs systems over a 48 h period at various pH settings. As shown in Figure 5a, the sample MS@GPTS/Cur NPs exhibited relatively fast and higher Cur release behavior, specifically ~26% at pH 7.4, and almost complete release was observed at pH 6.5, and 4.0, respectively. Around ~90% of the Cur was released from the MS@GPTS/Cur NPs in 8–12 h. The weak interaction between Cur molecules and the surface silanol and epoxy groups present in the MS@GPTS NPs caused the enhancement and bust release of Cur. Furthermore, at pH 4.0 conditions, the MS@GPTS/Cur NPs showed a burst release as compared to the pH 6.5 or 7.4 conditions, respectively, which could be attributed to the high solubility and possible electrostatic repulsive force between the protonated Cur molecules under acidic pH conditions [42].

For comparison, the release behavior of the free Cur was studied at different pH (pH 7.4, and 4) conditions, respectively. As observed in Figure 5c, the burst release was observed in 1 h and the Cur molecules are almost completely released in 2 h at the studied pH conditions. It is well known that the Cur is relatively stable in an acidic medium, but unstable at a pH >7. This could be due to the pH-induced changes in curcumin structure and physicochemical stability [43]. However, in this study, both pH 7.4 and pH 4 conditions, respectively, showed relatively burst release might be due to the fast diffusion of Cur molecules in the medium.

**Scheme 2:**
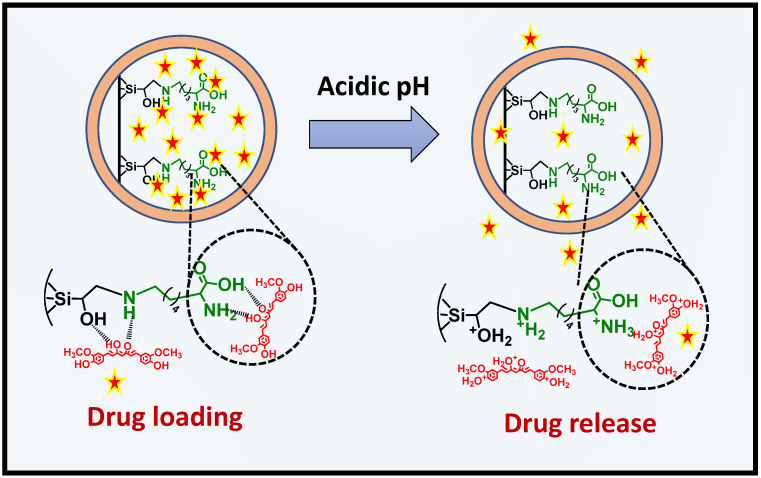
Schematic representation for the Cur loading, and pH-stimuli-responsive release from the MS@Lys/Cur NPs.

The L-lysine functionalized MS@Lys/Cur NPs, on the other hand, demonstrated significantly a slow-release behavior concerning the pH of the release medium (Figure 5b). In 48 h, only about ~20% of the loaded Cur molecules were released at physiological pH (pH 7.4), indicating that the loaded Cur molecules firmly interacted with the drug binding sites such as amine and carboxylic acid groups of the functionalized L-lysine groups in the MS@Lys NPs (Scheme 2). In contrast, at acidic pH (pH 6.5 and 4.0), the MS@Lys/Cur NPs released around ~65% and complete release of Cur in 48 h, respectively (Figure 5b). In the 24 h release period, cur release was about twice as high under pH 4.0 conditions as it was under pH 6.5 conditions (Table 2). The drug release studies demonstrated that the presence of amine and carboxylic acid groups on the surface of the MS@Lys/Cur NPs controlled the release of Cur effectively. The ζ-potential values of MS@Lys NPs might explain the Cur release behavior of MS/Lys/Cur NPs. According to the results of the ζ-potential investigation, the MS@Lys NPs have positive charges at pH 4.0, due to the presence of more amine groups of L-lysine units, which influences the electrostatic interaction or repulsion with Cur molecules in response to the medium pH. At higher pH, the Cur molecules become deprotonated resulting in a strong electrostatic interaction with the positively charged amine groups of L-lysine present on the surface of the MS@Lys NPs. This leads to a decreased Cur release at physiological pH (pH 7.4) [44].

In contrast, under acidic pH circumstances, the Cur molecules became protonated, resulting in an electrostatic repulsive force between the protonated Cur molecules and the protonated amine and carboxylic acid groups, resulting in a continuous release of Cur over time [45]. The prepared MS@Lys/Cur NPs’ pH-responsive release characteristic was further verified by assessing the Cur release by changing the pH of the release medium at a predetermined time point. As shown in Figure 5d, the Cur release from the MS@Lys/Cur NPs was monitored for the first 12 h by keeping the pH of the release medium at pH 7.4. During this release period, the Cur was released considerably slowly, with only around ~20% observed. Unlike naturally occurring curcuminoid mixtures (that contain curcumin, demethoxy-curcumin, and bisdemethoxy-curcumin), pure curcumin was considerably unstable to chemical degradation in alkaline aqueous solutions (pH ≥ 7.0) and tended to crystallize out of aqueous acidic solutions (pH < 7). These effects were attributed to changes in the molecular structure of curcumin under different pH conditions. Nevertheless, after 12 h, the pH of the release medium was adjusted to 4.0 using an acetate buffer. As shown in Figure 5d, the Cur release increased noticeably with time, with approximately ~70% of the Cur release seen in 24 h and roughly ~90% of the Cur release attained in the 48 h release period. Therefore, the prepared MS@Lys NPs could be used as a nanocarrier system for holding and sustained release of therapeutics in drug delivery applications.

### 3.3. In Vitro Cytocompatibility (MTT Assay) Study

**Figure 6:**
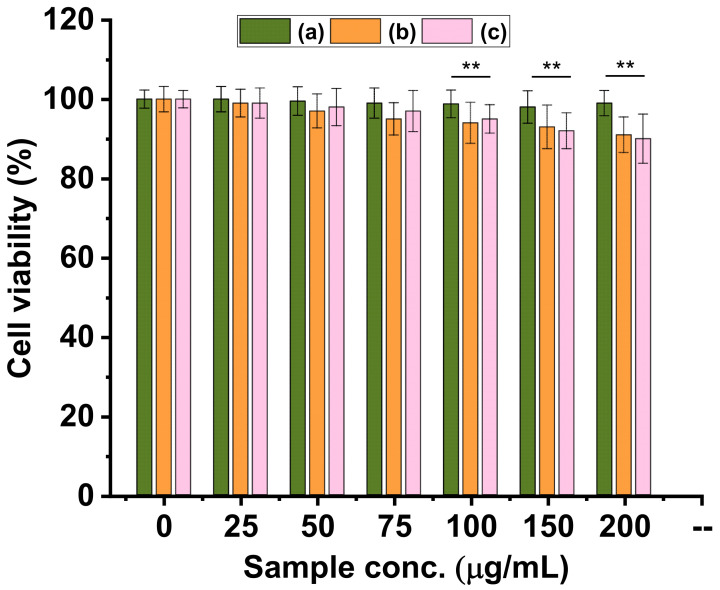
In vitro cytocompatibility of MDA-MB-231 cells exposed to (a) only control cells; (b) MS@GPTS NPs, and (c) MS@Lys NPs treated cells, respectively, at different sample concentrations. (**, highly significant *p* < 0.01).

The in vitro biocompatibility of the prepared MS@GPTS NPs and MS@Lys NPs, respectively, were tested in vitro at various sample concentrations (0–200 μg/mL) using the MTT (3-(4,5-dimethylthiazol-2-yl)-2,5-diphenyltetrazolium bromide) assay. Human breast cancer cell line MDA-MB-231 cells were used as a model cell line for these experiments, and they were exposed to MS@GPTS NPs and MS@Lys NPs without Cur loading at varied concentrations at 24 h incubation period. As shown in Figure 6, the cell viability of MDA-MB-231 cells treated with only MS@GPTS NPs and MS@Lys NPs without drug loading showed almost more than ~90% of the cells were in live condition at the tested sample concentrations. The observed results suggest that MS@GPTS NPs and MS@Lys NPs are both biocompatible.

**Figure 7:**
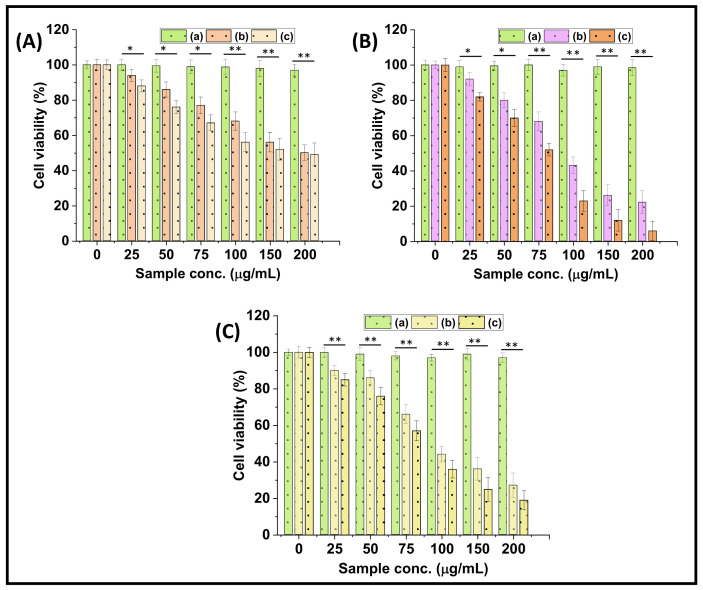
In vitro cytotoxicity of (**A**) MS@GPTS/Cur NPs; (**B**) MS@Lys/Cur NPs; respectively, treated to the MDA-MB-231 cells, at different sample concentrations. (**C**) In vitro cytotoxicity of only Cur molecules in DMSO exposed to the MDA-MB-231 cells at different concentrations. In Figure, (a): only control cells; (b): MDA-MB-231 cells treated at 24 h; and (c): MDA-MB-231 cells treated at 48 h. (*, significant *p* < 0.05; **, highly significant *p* < 0.01).

Furthermore, the cytotoxicity behavior of the Cur-loaded MS@GPTS/Cur NPs and MS@Lys/Cur NPs and free Cur, respectively, were tested in vitro at various sample concentrations (0–200 μg/mL) using the MTT assay analysis on MDA-MB-231 cell line. The cells were exposed to MS@GPTS/Cur NPs and MS@Lys/Cur NPs and free Cur, respectively, over two different incubation times (12 h and 24 h). As shown in Figure 7A, the MS@GPTS/Cur NPs demonstrated around ~50% cell toxicity during the incubation of 24 h and 48 h, respectively, even at higher sample concentrations of ~200 μg/mL. In contrast, the MS@Lys/Cur NPs treated cells at the sample concentration of ~200 μg/mL demonstrated around ~90% cell killing efficiency (Figure 7B). Moreover, the pure Cur-treated cells demonstrated around ~83% cell toxicity to MDA-MB-231 cells (Figure 7C). As compared to the MS@GPTS/Cur NPs, MS@Lys/Cur NPs, respectively, showed an enhanced cell-killing efficiency. This may be due to the reason that the percentage of Cur loaded into MS@Lys/Cur NPs is significantly higher (~68%) than the Cur loaded into the MS@GPTS/Cur NPs (approximately ~32%). Owing to the higher Cur loading into the MS@Lys/Cur NPs, the MS@Lys/Cur NPs showed enhanced cell toxicity to the MDA-MB-231 cells might be due to the slow and sustained release of Cur molecules from the mesopore channels of the MS@Lys/Cur NPs under the intracellular medium which induced more cells to death than the MS@GPTS/Cur NPs and pure Cur samples [46]. This could be explained by the fact that the MDA-MB-231 cells can take in more MS@Lys/Cur NPs when treated for 48 h, releasing more loaded Cur in a sustained manner than when treated for 24 h. Since nanoparticles may be internalized into cells via an intracellular uptake process, pure drugs can enter cells via endocytosis pathways [47]. According to these results (Figure 7), it is possible to infer that L-lysine functionalized MS@Lys NPs are effective for pH-responsive sustained drug delivery applications.

### 3.4. Cell Uptake Study

**Figure 8:**
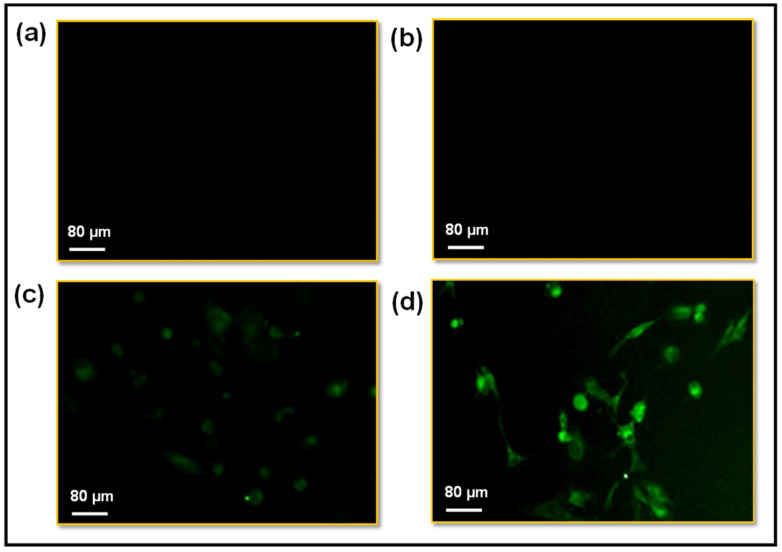
(**a**) Fluorescence microscopy images of MDA-MB-231 cells treated with (**a**) only control MDA-MB-231 cells; (**b**) cells treated only MS@Lys NPs without Cur loading; (**c**) cells treated with MS@Lys/Cur NPs for 1 h incubation; (**d**) cells treated with MS@Lys/Cur NPs for 5 h incubation, respectively. Scale bar = 80 μm.

The in vitro cell uptake behavior of MS@Lys NPs was studied using a fluorescence microscopic technique. As seen in Figure 8, after 5 h incubation, neither the reference cell nor the MDA-MB-231 cells treated with only MS@Lys NPs without Cur loading showed any detectable fluorescence (Figure 8b). MDA-MB-231 cells treated with Cur-loaded MS@Lys/Cur NPs, on the other hand, displayed green fluorescence in the cells, suggesting that the MS@/Lys/Cur NPs were taken up by the MDA-MB-231 cells and the loaded Cur was released from the MS@/Lys/Cur NPs. As shown in Figure 8c,d, the intensity of green fluorescence was increased in cells treated with MS@/Lys/Cur NPs for 5 h (Figure 8d) than in cells incubated for 1 h (Figure 8c). This suggests that when the incubation period was increased, more MS@/Lys/Cur NPs were taken up by the MDA-MB-231 cells, releasing the loaded drug inside the cells. The fluorescence study findings revealed that the prepared MS@Lys NPs are readily taken up by MDA-MB-231 cells, suggesting that they might be used for pH-responsive controlled drug delivery applications in cancer therapy.

### 3.5. In Vitro Hemocompatibility Study

**Figure 9:**
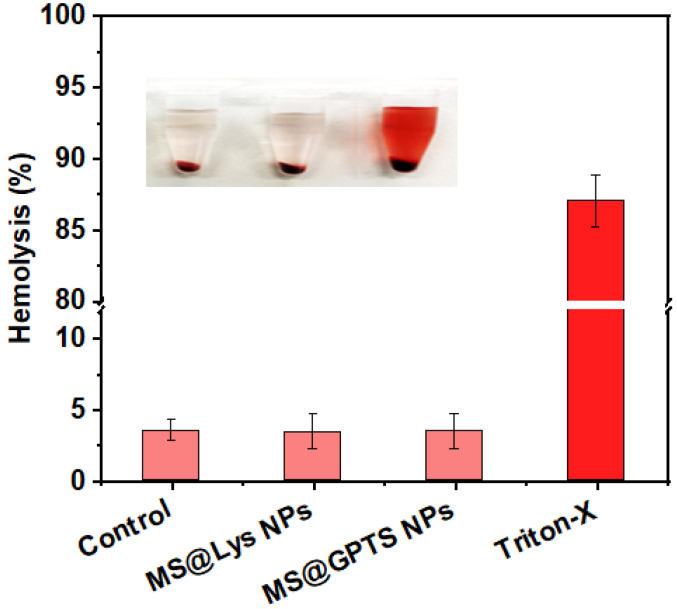
In vitro hemolysis behavior of the MS@Lys NPs and MS@GPTS NPs, respectively. Inset photographic images of control, MS@Lys NPs, and Triton-X, respectively.

The prepared MS@Lys NPs’ in vitro hemocompatibility was investigated using a hemolysis assay. The vial containing the supernatant of the control and MS@Lys NPs treated with RBCs was transparent and clear, as shown in Figure 9 (inset figure). The supernatant solution of the Triton-X treated RBCs, on the other hand, was cloudy and red in color due to the release of hemoglobin from the RBCs caused by the rupture of cell membranes when exposed to the Triton-X. 

Figure 9 shows that the MS@Lys NPs treated sample had a hemolysis rate of around 3.5%, which is below the permissible range (5%) [48]. For comparison, we have also examined the hemolysis behavior of the MS@GPTS NPs and the study results showed that the MS@GPTS NPs also showed a similar trend of hemolysis and the observed hemolysis rate of around ~3.6% (Figure 9). These findings support the fact that the prepared MS@GPTS NPs and MS@Lys NPs are hemocompatible.

## 4. Conclusions

In summary, mesoporous silica nanoparticles were functionalized with L-lysine, which includes both amine and carboxylic acid groups, after being integrated with 3-glycidoxypropyl trimethoxy silane in the mesopore walls and channels. The drug loading and pH-stimuli-responsive drug release characteristics of the prepared MS@Lys NPs were investigated at varied pH (pH 7.4, 6.5, and 4.0) settings using curcumin as a model anticancer agent. The drug loading efficiency study reveals that the prepared MS@Lys NPs can accommodate about ~68% of Cur due to the presence of surface-functionalized L-lysine groups in the MS@Lys NPs. Additionally, the pH-responsive drug release behavior of Cur-loaded MS@Lys/Cur NPs in acidic pH settings suggests that MS@Lys NPs might be used as pH-stimuli-responsive drug carriers. Additionally, the results of the in vitro cytocompatibility study show that the MS@Lys NPs are biocompatible and hemocompatible, and can be efficiently internalized into MDA-MB-231 cells. All of the experimental results suggest that MS@Lys NPs might be used for pH-responsive drug delivery applications in cancer therapy.

## Figures and Tables

**Table 1:** Mesoporous structural properties of the MS@GPTS NPs and MS@Lys NPs.

Sample	Surface Area (m^2^/g)	Pore Size (nm)	Pore Volume (cm^3^/g)	Cur Loading Efficiency (%)
MS@GPTS NPs	642 ± 0.5	3.6 ± 0.4	0.312 ± 0.67	~32 ± 1.2
MS@Lys NPs	527 ± 0.4	3.3 ± 0.6	0.236 ± 0.53	~68 ± 1.1

**Table 2:** Cur release from the MS@GPTS/Cur NPs and MS@Lys/Cur NPs, respectively at different pH conditions.

pH	Cur Release (%)	
	MS@GPTS/Cur NPs	MS@Lys/Cur NPs
7.4	26 ± 1.67	20 ± 1.6
6.5	100 ± 2.5	65 ± 2.2
4.0	100 ± 2.5	100 ± 1.9

## Data Availability

The data presented in this study are available on request from the corresponding author.
